# Supplementary material for: The Systems Biology Research Tool: evolvable open-source software
Source: BMC Syst Biol. 2008 Jun 29;2:55. doi: 10.1186/1752-0509-2-55 (PMC2446383; doi:10.1186/1752-0509-2-55)
Supplement: Additional file 1 — SBRT Archive. An archive of the current version of the Systems Biology Research Tool. [file 1752-0509-2-55-S1.zip › sbrt-1.4.0/doc/developers_guide/api/sbrt/shell/mng/stat/CorrelationManager.html]

CorrelationManager


|  |  |  |
| --- | --- | --- |
| **Overview** \| **Package** \| **Class** \| **Use** \| **Tree** \| **Deprecated** \| **Index** \| **Help** | |  |
| PREV CLASS   **NEXT CLASS** | **FRAMES**    **NO FRAMES**     **All Classes** |  |
| SUMMARY: NESTED \| FIELD \| CONSTR \| METHOD | DETAIL: FIELD \| CONSTR \| METHOD |  |


---


## sbrt.shell.mng.stat Class CorrelationManager

```
java.lang.Object
  sbrt.shell.mng.AbstractProcessManager
      sbrt.shell.mng.stat.CorrelationManager
```

**All Implemented Interfaces:**: KernelProcessManager<java.lang.String,java.lang.String>, ProcessManager<java.lang.String,java.lang.String>

---

``` public class CorrelationManager extends AbstractProcessManager implements KernelProcessManager<java.lang.String,java.lang.String> ```

This class is used to manage correlation processes.

**Author:**
:   This class was written and documented by Jeremiah Wright while in the
    Wagner lab.

---

| **Field Summary** | |
| --- | --- |
| `static java.lang.String` | `CORR_COEFF`             The keyword used to denote the type of correlation coefficient to be computed. |
| `static java.lang.String` | `KENDALLS_TAU`             The value used to denote a Kendall's tau correlation coefficient. |
| `static UnmodifiableSet<java.lang.String>` | `optionalKeywords`             The set of keywords understood, but not required, by this process manager. |
| `static java.lang.String` | `PEARSONS_R`             The value used to denote a Pearson's r correlation coefficient. |
| `static UnmodifiableSet<java.lang.String>` | `requiredKeywords`             The set of keywords required by this process manager. |
| `static java.lang.String` | `SPEARMANS_RHO`             The value used to denote a Spearman's rho correlation coefficient. |
| `static java.lang.String` | `X_FILE_NAME`             The keyword used to denote the name of the file containing the x values. |
| `static java.lang.String` | `Y_FILE_NAME`             The keyword used to denote the name of the file containing the y values. |

**Fields inherited from class sbrt.shell.mng.AbstractProcessManager**

`ALGORITHM, COMPLETED, DATA_HEADERS, DEV_NULL, ELAPSED_TIME, FILE_FORMAT, INPUT_FILE, INPUT_FILE_FORMAT, INPUT_FILE_NAME_FILE, ITERATIONS, OUTPUT_FILE_FORMAT, OUTPUT_FILE_NAME, OUTPUT_FILE_NAME_FILE, PERCENT, PERCENT_COMPLETED, SEED, ZERO_CUTOFF`


**Constructor Summary**

`CorrelationManager()`


| **Method Summary** | |
| --- | --- |
| `void` | `execute(java.io.PrintWriter out)`             Executes the process this process manager controls and writes the results to the provided print writer. |
| `UnmodifiableSet<java.lang.String>` | `getOptionalKeys()`             Returns the set of keywords understood, but not required, by this process manager. |
| `CorrelationAnalysis<java.lang.String,RCorrelator.Result>` | `getProcess()`             Returns the process controlled by this process manager. |
| `UnmodifiableSet<java.lang.String>` | `getRequiredKeys()`             Returns the set of keywords required by this process manager. |
| `static UnmodifiableMap<java.lang.String,java.lang.Double>` | `getVector(java.lang.String fileName)`             Parses the file with the provided name and returns the vector it contains. |
| `static UnmodifiableMap<java.lang.String,java.lang.Double>` | `getVector(java.lang.String fileName, java.io.PrintWriter out)`             Parses the file with the provided name, prints a status message to the provided print writer, and returns the vector the file contains. |
| `static UnmodifiableMap<java.lang.String,java.lang.Double>` | `getXValues(ManagerInput<java.lang.String,java.lang.String> input, java.io.PrintWriter out)`             Returns the parsed value for `X_FILE_NAME` in the provided input and prints a descriptive message to the provided print writer. |
| `static UnmodifiableMap<java.lang.String,java.lang.Double>` | `getYValues(ManagerInput<java.lang.String,java.lang.String> input, java.io.PrintWriter out)`             Returns the parsed value for `Y_FILE_NAME` in the provided input and prints a descriptive message to the provided print writer. |
| `static ApplicationException` | `interpret(JriException e)`             Wraps the provided JRI exception as an application exception. |
| `static RCorrelator<java.lang.String>` | `parseCorrelator(java.lang.String coeff)`             Returns the correlation computer corresponding to the provided string value. |
| `void` | `setInput(ManagerInput<java.lang.String,java.lang.String> input, java.io.PrintWriter out)`             Uses the provided input to prepare this process manager for execution. |
| `static void` | `startR(java.io.PrintWriter out)`             Starts the R engine and writes an informative message to the provided print writer. |
| `static void` | `writeIntersection(java.lang.String fileName, java.util.Map<java.lang.String,java.lang.Double> xValues, java.util.Map<java.lang.String,java.lang.Double> yValues, java.io.PrintWriter out)`             Writes the intersection of the provided vectors to a file with the specified name. |

**Methods inherited from class sbrt.shell.mng.AbstractProcessManager**

`check, closeFile, closeRm, execute, getFileFormat, getFileFormat, getFileNameSource, getIterations, getSeed, getTextFileFormat, getTextFileFormat, getZeroCutoff, getZeroCutoff, parseInt, parseLong, printProgress, printProgress, printProgress, runProcess, setInput`

**Methods inherited from class java.lang.Object**

`clone, equals, finalize, getClass, hashCode, notify, notifyAll, toString, wait, wait, wait`

**Methods inherited from interface sbrt.shell.mng.ProcessManager**

`check, execute, setInput`

**Field Detail**

### CORR\_COEFF

```
public static final java.lang.String CORR_COEFF
```

:   The keyword used to denote the type of correlation coefficient to be
    computed.

    **See Also:**: Constant Field Values

---


### KENDALLS\_TAU

```
public static final java.lang.String KENDALLS_TAU
```

:   The value used to denote a Kendall's tau correlation coefficient.

    **See Also:**: Constant Field Values

---


### PEARSONS\_R

```
public static final java.lang.String PEARSONS_R
```

:   The value used to denote a Pearson's r correlation coefficient.

    **See Also:**: Constant Field Values

---


### SPEARMANS\_RHO

```
public static final java.lang.String SPEARMANS_RHO
```

:   The value used to denote a Spearman's rho correlation coefficient.

    **See Also:**: Constant Field Values

---


### X\_FILE\_NAME

```
public static final java.lang.String X_FILE_NAME
```

:   The keyword used to denote the name of the file containing the x values.

    **See Also:**: Constant Field Values

---


### Y\_FILE\_NAME

```
public static final java.lang.String Y_FILE_NAME
```

:   The keyword used to denote the name of the file containing the y values.

    **See Also:**: Constant Field Values

---


### requiredKeywords

```
public static final UnmodifiableSet<java.lang.String> requiredKeywords
```

:   The set of keywords required by this process manager.

---


### optionalKeywords

```
public static final UnmodifiableSet<java.lang.String> optionalKeywords
```

:   The set of keywords understood, but not required, by this process
    manager.


**Constructor Detail**

### CorrelationManager

```
public CorrelationManager()
```


**Method Detail**

### getVector

```
public static UnmodifiableMap<java.lang.String,java.lang.Double> getVector(java.lang.String fileName)
```

:   Parses the file with the provided name and returns the vector it
    contains. The file must be a row-oriented map file whose variable-value
    pairs are parsable using `EqualsMapFormatV1`. The variables are
    not parsed further, and the values are parsed using
    `DoubleFormatV1`.

    :   **Parameters:**: `fileName` - the name of the file to be read. **Returns:**: variables mapped to their corresponding value.

---


### getVector

```
public static UnmodifiableMap<java.lang.String,java.lang.Double> getVector(java.lang.String fileName,
                                                                           java.io.PrintWriter out)
```

:   Parses the file with the provided name, prints a status message to the
    provided print writer, and returns the vector the file contains. The file
    must be a row-oriented map file whose variable-value pairs are parsable
    using `EqualsMapFormatV1`. The variables are not parsed further,
    and the values are parsed using `DoubleFormatV1`.

    :   **Parameters:**: `fileName` - the name of the file to be read.: `out` - the print writer to which a status message will be written. **Returns:**: variables mapped to their corresponding value.

---


### getXValues

```
public static UnmodifiableMap<java.lang.String,java.lang.Double> getXValues(ManagerInput<java.lang.String,java.lang.String> input,
                                                                            java.io.PrintWriter out)
```

:   Returns the parsed value for `X_FILE_NAME` in the provided input
    and prints a descriptive message to the provided print writer.

    :   **Parameters:**: `input` - the input whose `X_FILE_NAME` value is to be parsed.: `out` - the print writer to which a message is printed. **Returns:**: variables mapped to their corresponding value. **See Also:**: `getVector(String, PrintWriter)`

---


### getYValues

```
public static UnmodifiableMap<java.lang.String,java.lang.Double> getYValues(ManagerInput<java.lang.String,java.lang.String> input,
                                                                            java.io.PrintWriter out)
```

:   Returns the parsed value for `Y_FILE_NAME` in the provided input
    and prints a descriptive message to the provided print writer.

    :   **Parameters:**: `input` - the input whose `Y_FILE_NAME` value is to be parsed.: `out` - the print writer to which a message is printed. **Returns:**: variables mapped to their corresponding value. **See Also:**: `getVector(String, PrintWriter)`

---


### writeIntersection

```
public static void writeIntersection(java.lang.String fileName,
                                     java.util.Map<java.lang.String,java.lang.Double> xValues,
                                     java.util.Map<java.lang.String,java.lang.Double> yValues,
                                     java.io.PrintWriter out)
```

:   Writes the intersection of the provided vectors to a file with the specified
    name.

    :   **Parameters:**: `fileName` - the desired name of the output file.: `xValues` - the x values.: `yValues` - the y values.: `out` - the print writer to which descriptive messages will be written.

---


### parseCorrelator

```
public static RCorrelator<java.lang.String> parseCorrelator(java.lang.String coeff)
```

:   Returns the correlation computer corresponding to the provided string
    value.

    :   **Parameters:**: `coeff` - a string equal to KENDALLS\_TAU, SPEARMANS\_RHO, or PEARSONS\_R. **Returns:**: the correlation computer corresponding to the provided string value. **Throws:**: `ApplicationException` - if the provided string does not equal KENDALLS\_TAU, SPEARMANS\_RHO, or PEARSONS\_R.

---


### startR

```
public static void startR(java.io.PrintWriter out)
```

:   Starts the R engine and writes an informative message to the provided
    print writer.

    :   **Parameters:**: `out` - the print writer to which a message will be written.

---


### interpret

```
public static ApplicationException interpret(JriException e)
```

:   Wraps the provided JRI exception as an application exception.

    :   **Parameters:**: `e` - the JRI exception. **Returns:**: an application exception.

---


### getRequiredKeys

```
public UnmodifiableSet<java.lang.String> getRequiredKeys()
```

:   Returns the set of keywords required by this process manager.

    :   **Specified by:**: `getRequiredKeys` in interface `ProcessManager<java.lang.String,java.lang.String>`
    :   **Returns:**: the set of keywords required by this process manager.

---


### getOptionalKeys

```
public UnmodifiableSet<java.lang.String> getOptionalKeys()
```

:   Returns the set of keywords understood, but not required, by this process
    manager.

    :   **Specified by:**: `getOptionalKeys` in interface `ProcessManager<java.lang.String,java.lang.String>`
    :   **Returns:**: the set of keywords understood, but not required, by this process manager.

---


### getProcess

```
public CorrelationAnalysis<java.lang.String,RCorrelator.Result> getProcess()
```

:   Returns the process controlled by this process manager.

    :   **Specified by:**: `getProcess` in interface `KernelProcessManager<java.lang.String,java.lang.String>`
    :   **Returns:**: the process controlled by this process manager.

---


### setInput

```
public void setInput(ManagerInput<java.lang.String,java.lang.String> input,
                     java.io.PrintWriter out)
```

:   Uses the provided input to prepare this process manager for execution. As
    the input is processed, informative messages are printed to the provided
    print writer.

    :   **Specified by:**: `setInput` in interface `ProcessManager<java.lang.String,java.lang.String>`
    :   **Parameters:**: `input` - all of the information required to prepare this process manager for execution.: `out` - the print writer to which all messages will be printed.

---


### execute

```
public void execute(java.io.PrintWriter out)
```

:   Executes the process this process manager controls and writes the results
    to the provided print writer.

    :   **Specified by:**: `execute` in interface `ProcessManager<java.lang.String,java.lang.String>`
    :   **Parameters:**: `out` - the print writer where the results will be printed. **Throws:**: `java.lang.IllegalStateException` - if no input was provided to this process manager.


---


|  |  |  |
| --- | --- | --- |
| **Overview** \| **Package** \| **Class** \| **Use** \| **Tree** \| **Deprecated** \| **Index** \| **Help** | |  |
| PREV CLASS   **NEXT CLASS** | **FRAMES**    **NO FRAMES**     **All Classes** |  |
| SUMMARY: NESTED \| FIELD \| CONSTR \| METHOD | DETAIL: FIELD \| CONSTR \| METHOD |  |


---
